# Navigating treatment approaches for presumed ESBL-producing infections

**DOI:** 10.1093/jacamr/dlaa111

**Published:** 2021-02-24

**Authors:** Pranita D Tamma, Amy J Mathers

**Affiliations:** 1 Johns Hopkins University School of Medicine, Department of Pediatrics, Baltimore, MD, USA; 2 University of Virginia, Department of Medicine and Pathology, Charlottesville, VA, USA

## Abstract

ESBL-producing Enterobacterales (ESBL-E) remain a significant global threat. In several regions of the world, ESBLs are produced by over half of *Escherichia coli* or *Klebsiella pneumoniae* infections, contributing to significant morbidity and mortality. Though it is accepted that carbapenems are effective for the treatment of invasive ESBL-E infections, controversy remains as to whether carbapenem alternatives can be considered in select cases. Indiscriminate carbapenem use for the treatment of ESBL-E infections will likely further the international antimicrobial resistance crisis, underscoring the importance of investigating the role of non-carbapenem options. In this issue of *JAC-Antimicrobial Resistance*, we present a PRO/CON debate exploring whether carbapenems are necessary for all infections caused by ceftriaxone-resistant Enterobacterales.

ESBL-producing Enterobacterales (ESBL-E) infections are occurring at alarming rates on a global scale. A number of countries from diverse regions of the world report that more than half of *Escherichia coli* isolates are ceftriaxone resistant (i.e. presumed to be ESBL-producing), including Mexico (58%), China (64%), India (77%), Russia (73%) and Nigeria (77%).^1^ The CDC estimate that the incidence of ESBL-E infections in the USA increased by 53% from 2012 through 2017.^2^ In contrast, the incidence of carbapenem-resistant Enterobacterales in the USA has plateaued during this same period. Reasons for the success of ESBL-E include horizontal transfer of mobile genetic elements harbouring ESBL genes (e.g. IncF plasmids), successful bacterial clones (e.g. *E. coli* ST131), ESBL gene transfer from animal products, antimicrobial overuse, poor sanitation and human travel and migration. The relatively high mortality associated with ESBL-producing infections—upwards of 30% in some studies—is in part due to delays in initiating effective antimicrobial therapy.^3^

Carbapenems are effective against ESBL-E infections. However, the global emergence of carbapenem-resistant organisms has prompted investigations into opportunities for carbapenem-sparing regimens to preserve activity of the carbapenem class. A growing body of observational studies prior to the publication of the MERINO trial suggested that use of piperacillin/tazobactam administered as 4500 mg every 6 h led to similar clinical outcomes to carbapenems.^4^ This was particularly the case with bloodstream infections (BSI) due to ‘low bacterial burden’ sources such as urinary or biliary sites, in non-critically ill patients, and organisms with low piperacillin/tazobactam MICs.^5^ Several of these studies were large, multicentre studies, but despite the best attempts by investigators, risk of bias in treatment assignment remained a concern and there were likely inherent differences between the patients treated with piperacillin/tazobactam and those treated with carbapenem therapy.^5^ These observational studies brought a collective sigh of relief to the infectious diseases community as there had been apprehension regarding the consequences of carbapenem overuse that would occur in response to the rising rates of ESBL-E infections.

This all changed after the publication of the MERINO trial. The MERINO trial was a pragmatic, randomized trial that compared the clinical outcomes of patients with ceftriaxone non-susceptible *E. coli* or *Klebsiella* spp. BSI treated with piperacillin/tazobactam versus meropenem.^4^ Although the intention was a non-inferiority study, it quite decisively showed that patients receiving piperacillin/tazobactam had a higher likelihood of not surviving the ensuing 30 days compared with patients receiving carbapenem therapy. The results were striking and resulted in premature termination of the study. The MERINO trial did not have sufficient power for robust subgroup analyses, but the investigators found that regardless of the source of bacteraemia, the severity of illness or host immune status, piperacillin/tazobactam appeared to perform inferiorly to carbapenem therapy for the treatment of ESBL-E BSI.

Appreciating the inaccuracies of piperacillin/tazobactam antimicrobial susceptibility testing with the automated susceptibility platforms or disc diffusion methods used at most participating trial sites, the MERINO investigators conducted a post-hoc analysis limited to patients with *E. coli* or *Klebsiella* spp. with piperacillin/tazobactam MICs of ≤16 mg/L according to the results of reference broth microdilution.^6^ The increase in mortality was more modest when re-analysing results—the absolute difference in increased risk of 30 day mortality with piperacillin/tazobactam was 5% (95% CI −1% to 10%). While technically no longer significant, the results likely will still give many clinicians pause in confidently prescribing piperacillin/tazobactam for ESBL-producing infections, particularly because, in the real world, inaccuracies in obtaining piperacillin/tazobactam MICs exist in most hospital systems. Only a shrinking minority of clinical microbiology laboratories are using reference broth microdilution to derive piperacillin/tazobactam MICs and thus there will be continued concerns for underestimating piperacillin/tazobactam resistance.

In this issue of *JAC-Antimicrobial Resistance*, invited international leaders undertake a PRO/CON debate regarding treatment approaches for presumed ESBL-E infections. Paterson and colleagues^7^ rest on the merits of the MERINO trial and argue in favour of carbapenem therapy for these infections. Rodríguez-Baño and colleagues^8^ raise important rebuttals concerning the MERINO trial and support the position that carbapenem therapy is not always necessary to treat ESBL-producing infections. Carefully weighing both arguments will help clinicians navigate the management of these increasingly common infections.

## Funding

This work was supported by funding from the National Institutes of Health K23-AI127935 awarded to P.D.T.

## Transparency declarations

P.D.T. has none to declare. A.J.M. has served as an advisor for Rempex and as a consultant/advisory panel member for Qpex Biopharma, Accelerate Diagnostics, VenatoRX and Antimicrobial Resistance Services, Inc.
